# Serum miRNA expression profile in responders to acupuncture treatment for diminished ovarian reserve: a study protocol for a nested case-control study

**DOI:** 10.3389/fendo.2026.1972018

**Published:** 2026-10-07

**Authors:** Dongxue An, Li Yang, Jiashan Li, Tian Hang, Lingyu Qi, Jiaxi Meng, Chenchen Su, Quanying Zhang, Ge Lu, Binbin Wang, Yigong Fang, Huanfang Xu

**Affiliations:** 1 Institute of Acupuncture and Moxibustion, China Academy of Chinese Medical Sciences, Beijing, China; 2 Center for Genetics, National Research Institute for Family Planning, Beijing, China

**Keywords:** acupuncture, diminished ovarian reserve, microRNA, nested case-control study, study protocol

## Abstract

**Objective:**

Diminished ovarian reserve (DOR) leads to subfertility, infertility, and premature menopause, significantly compromising women’s reproductive health. Although clinical evidence supports the effectiveness of acupuncture in improving ovarian reserve, treatment responses vary considerably among DOR patients, and the factors associated with favorable outcomes remain unclear. Serum microRNAs may serve as potential biomarkers for distinguishing acupuncture responders, warranting further investigation.

**Design and methods:**

This prospective nested case-control study will enroll women with DOR who undergo acupuncture treatment. Treatment response will be evaluated based on changes in anti-Müllerian hormone (AMH) levels, antral follicle count (AFC), serum follicle stimulating hormone (FSH) levels and clinical pregnancy. According to their response, participants demonstrating greater improvement will be assigned to the experimental group (responders), while non-responders will be matched in a 1:1 ratio to form the control group. Serum microRNA profiles from both groups will be analyzed using high-throughput sequencing to identify differentially expressed microRNAs. Candidate microRNAs will subsequently be validated via real-time quantitative PCR (RT-qPCR), and receiver operating characteristic (ROC) curve analysis will be performed to evaluate their predictive value for acupuncture treatment response.

**Discussion:**

This is the first clinical study to characterize the serum miRNA expression profiles of DOR patients who benefit from acupuncture. The findings are expected to provide evidence supporting the precise identification of acupuncture-responsive individuals within this patient population.

**Study protocol registration:**

https://itmctr.ccebtcm.org.cn/mgt/project/view/-4614215526207679163, identifier ITMCTR2025000004.

## Introduction

With advancing age, the finite ovarian follicular pool undergoes progressive depletion through oocyte atresia, culminating in menopause, with age being an independent risk factor for female reproductive capacity (1). Premature depletion of ovarian reserve that is inconsistent with chronological age, termed diminished ovarian reserve (DOR), refers to a reduction in both the quantity and quality of oocytes. Clinically, DOR is characterized by decreased anti-Müllerian hormone (AMH) levels and antral follicle count (AFC), alongside elevated basal follicle-stimulating hormone (FSH) levels (2, 3). This condition compromises ovarian function and diminishes fertility, leading to poor responses to controlled ovarian stimulation, lower pregnancy success rates in both natural conception and assisted reproductive technology (4, 5), increased risks of pregnancy-related comorbidities and complications, and adverse neonatal outcomes (6). With a prevalence of approximately 10% (7), the incidence of DOR is rising annually and increasingly affecting younger women, driven by escalating socioeconomic and occupational pressures, worsening environmental pollution, dietary changes, and delayed childbearing.

The pathogenesis of DOR remains poorly understood, and its clinical management is both challenging and frequently protracted. Despite these difficulties, patients typically exhibit a strong motivation to achieve fertility. Currently, no definitive therapeutic strategies exist to effectively restore ovarian function (3, 8). Nevertheless, accumulating clinical evidences suggest that acupuncture may promote ovarian function, including normalization of aberrant sex hormone levels (i.e., reduced FSH and Luteinizing Hormone [LH], elevated estradiol [E_2_]), increase in AFC and improvement in pregnancy outcomes (9–11). Systematic reviews and meta-analyses have further underscored the significant clinical promise of acupuncture in improving hormonal profiles and AFC among DOR patients (12, 13). However, marked inter-individual variability in treatment response has been observed in clinical practice, and it remains unclear which DOR patients are most likely to benefit from acupuncture. Considering the strong desire for treatment, the extended duration of therapy, and the associated economic strain, identifying predictors of acupuncture responsiveness is clinically valuable.

MicroRNAs (miRNAs) are a class of endogenous, non-coding RNAs, approximately 20–25 nucleotides in length, and always function as potent post-transcriptional regulators of gene expression. Widely distributed in human tissues and body fluids, they negatively regulate approximately one-third of protein-coding genes and are critically involved in cell proliferation, differentiation, apoptosis, immune response, and angiogenesis (14, 15). Within the ovary, miRNAs modulate ovarian function and contribute to DOR pathogenesis by targeting specific genes. They have been implicated in multiple ovarian processes, including follicular development (e.g., miR-503, miR-21, miR-142-3p) (16), follicular-luteal transition (e.g., miR-21) (17), granulosa cell apoptosis, and sex hormone metabolism. Serum miRNA expression profiles distinguish patients with premature ovarian failure (POF) from those with normal ovarian reserve (18). For example, miR-22-3p, a modulator of FSH responsiveness, is significantly downregulated in the serum of POF patients relative to controls (18). These differentially expressed miRNAs may regulate granulosa cell proliferation and apoptosis via diverse signaling pathways. Additionally, miR-27a and miR-23a have shown potential as quantitative biomarkers for POF prognosis (19). Collectively, serum miRNAs represent promising candidate biomarkers for predicting acupuncture efficacy and for characterizing the subset of patients most likely to benefit from this therapy.

This prospective nested case-control study will enroll DOR patients receiving acupuncture. Treatment response will be assessed to identify acupuncture responders, defined as patients who show greater improvement in ovarian reserve, and these responders will then be matched in a 1:1 ratio with non-responders. High-throughput sequencing will be performed to screen for differentially expressed serum miRNAs, followed by validation using real-time quantitative PCR (RT-qPCR). Receiver operating characteristic (ROC) curve analysis will be used to evaluate the predictive value of candidate miRNAs for acupuncture response. The results are expected to delineate the serum miRNA expression profiles characteristic of acupuncture-responsive DOR patients and to facilitate the precise identification of individuals likely to benefit from this intervention.

## Methods

### Study design and setting

This prospective, single-center, nested case-control study will be conducted at the Acupuncture and Moxibustion Hospital of the China Academy of Chinese Medical Sciences. Eligible DOR patients will receive 12 weeks of acupuncture treatment. The trial will be conducted in accordance with the Declaration of Helsinki. It has been approved by the Ethics Committee of the China Academy of Chinese Medical Sciences (approval number: S2024-04-23-1). Written informed consent will be obtained from participants prior to enrolment. This protocol has been registered at the International Traditional Medicine Clinical Trial Registry (ITMCTR2025000004). The trial flow is shown in **Figure 1**. The schedule of enrolment, interventions and assessments of the trial is provided in **Table 1**.

**Figure 1:**
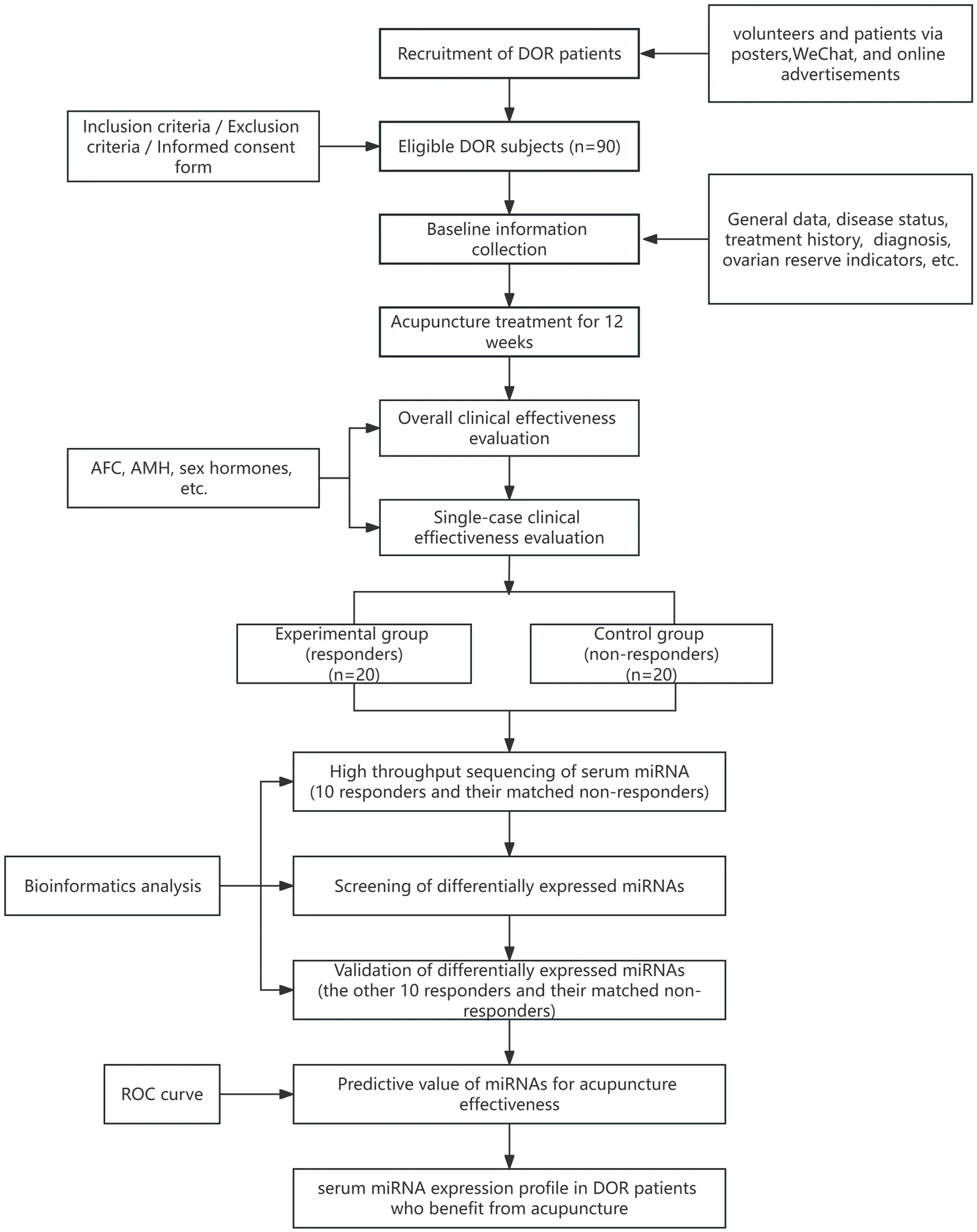
Flow diagram of the study design.

**Table 1:** Study timeline and assessment schedule.

Time Point	Items	Week 0	Week 4	Week 8	Week 12
Enrolment	Informed consent	×			
	Inclusion/Exclusion criteria	×			
	Baseline information	×			
Intervention	Acupuncture		×	×	×
Assessments	Serum microRNA	×			×
	AFC	×			
	Serum levels of basal FSH, LH, and E_2_	×			×
	Serum level of AMH	×			×
	Clinical pregnancy				×
	AEs	×	×	×	×

AFC, antral follicle count; FSH, follicle-stimulating hormone; LH, luteinizing hormone; Estradiol, E_2_; AMH, anti-Müllerian hormone; AEs, adverse events.

### Study participants

A total of 90 women with DOR will be recruited via posters, WeChat, or online advertisements. All participants will be given a complete and comprehensive explanation of the study purpose, procedure, benefits, risks, etc.

Women aged 22–40 years, having a serum level of AMH < 1.2 ng/mL and AFC < 5, and willing to undergo acupuncture will be invited to participate in the study.

Participants will be excluded if they had any of the following conditions: basal serum level of FSH ≥25 IU/L; diseases affecting ovulation, such as polycystic ovary syndrome, uncontrolled hyperprolactinemia, hypothyroidism, etc.; premature ovarian aging caused by iatrogenic factors (e.g., pelvic surgery, radiotherapy/chemotherapy, uterine artery embolization, medications such as hormones, immunosuppressants); abnormal karyotype; comorbid severe cardiovascular, hepatic, renal, malignant tumor, hematopoietic system, or psychiatric diseases.

### Intervention

A clinically established acupuncture protocol for improving ovarian function will be used. This protocol has been used for over ten years in our team with verified effect (20). It consists of two sets of acupoints for different body positions (**Figure 2**). Set 1 (supine position): GV20 (Baihui), GV24 (Shenting), GB13 (Benshen), CV12 (Zhongwan), ST25 (Tianshu), CV4 (Guanyuan), KI12 (Dahe), EX-CA1 (Zigong), ST36 (Zusanli), SP6 (Sanyinjiao), and LR3 (Taichong). Set 2: BL23 (Shenshu), BL33 (Zhongliao), and KI3 (Taixi). The two point sets will be used alternately, starting with Set 1. All acupoints will be located according to the WHO Standard Acupuncture Point Locations in the Western Pacific Region (21). Acupoints on the head will be needled transversely to a depth of 10–20 mm. BL33 will be needled obliquely downward into the third posterior sacral foramina for 50–60 mm. The remaining acupoints located on the trunk and limbs will be perpendicularly needled for 30–40 mm. Following needle insertion, gentle manipulation of lifting, thrusting, or twisting can be applied to the acupoints to achieve the desired needle sensation. Each acupuncture session will last 20 minutes. Treatment will be administered 3 times per week, for a total of 12 weeks (36 sessions).

**Figure 2:**
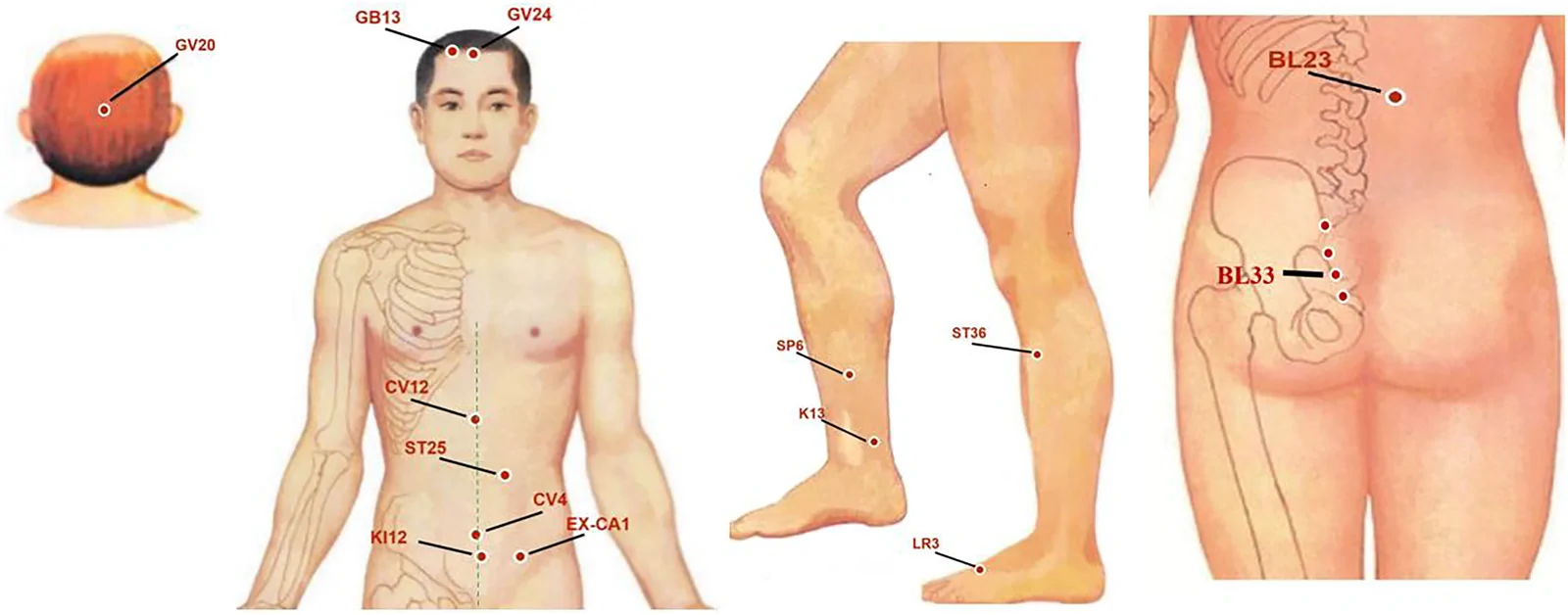
Acupoint location diagram.

### Data collection and management

A traceable electronic data recording system will be used to collect treatment data, which includes but not limites to demographic data, chief complaint, history of present illness, menstrual history, marital and obstetric history, acupuncture treatment, laboratory examination and adverse events record.

### Effectiveness evaluation

#### Overall acupuncture effectiveness assessment

The primary outcome is the change from baseline in AFC at week 12. Secondary outcomes include AMH, FSH, the ratio of FSH/LH, E_2_ and clinical pregnancy at week 12. The measurement of AFC, FSH, LH and E_2_ should be performed on days 2–4 of the menstrual cycle. Adverse events will be monitored and detailed recorded throughout the trial.

#### Single case effectiveness assessment

Based on the following criteria, the effectiveness of acupuncture for each participant will be evaluated to determine whether it is effective or ineffective, as well as the degree of effectiveness.

##### (1) Criteria for determining acupuncture-responsive cases

If after 12 weeks of acupuncture treatment, AFC, FSH, and AMH all show improvement compared to baseline, and meet any one of the following criteria, the case can be judged as clinically responsive: ① AFC > 7 or an increase of ≥2; ② Baseline FSH < 10 IU/L; ③ AMH ≥ 1.2 ng/ml; ④ clinical pregnancy.

Basal FSH and AFC should both be measured on days 2–5 of the menstrual cycle. AFC will be assessed by transvaginal ultrasonography, counting antral follicles 2–10 mm in diameter in both ovaries, with bilateral and total antral follicle counts recorded separately. To minimize inter-operator variability, all AFC measurements will be performed by a designated sonographer who has at least 2 years of experience in gynecological ultrasonography and has completed standardized training for this study. Clinical pregnancy is considered at the corresponding tier only for patients who are eligible for pregnancy assessment. Although clinical pregnancy is a more important endpoint, it cannot be mandatory in this 12-week trial, as enrollment does not restrict reproductive plans or contraceptive status. Therefore, AMH, AFC, and FSH serve as mandatory outcomes, while clinical pregnancy is exploratory for those planning to conceive.

##### (2) Selection of 10 patients with best therapeutic effect

For genome-wide screening of differentially expressed miRNAs, extreme phenotype-based sampling is a well-established biomarker discovery strategy and has been widely used in high-throughput genomics, proteomics, and metabolomics studies. Accordingly, we will apply extreme sampling to select 10 patients with the best therapeutic effect (experimental group) and 10 matched non-responders (control group) for high-throughput sequencing, aiming to maximize sensitivity, minimize false negatives, and identify candidate miRNAs with the greatest predictive potential.

To ensure objectivity and reproducibility, a composite ranking scheme will be applied that integrates the clinical importance of the ovarian reserve indicators with the number of items showing effective improvement. The indicators are hierarchically ranked by clinical importance as follows: clinical pregnancy > AFC/AMH > FSH. Based on this hierarchical framework and the number of effectively improved items, the top 10 responders will be identified. This scheme was developed in consultation with clinical experts.

##### (3) Criteria for determining non-responsive cases

If after treatment, indicators such as AMH, AFC, and FSH show no improvement or worsen compared to baseline, the case can be judged as non-responsive to acupuncture.

##### (4) Matching of responder and non-responder

Non-responsive cases will be matched 1:1 with the cases in the experimental group based on baseline age, AMH, AFC, and baseline FSH levels, and serve as the control group. Other potential confounding factors, such as duration of infertility, body mass index (BMI), and previous in vitro fertilization (IVF) failure history, will not be included given the limited sample size.

### Screening and validation of differentially expressed serum miRNAs

Serum will be collected at baseline and after 12 weeks of acupuncture. Peripheral blood will be drawn after overnight fasting, clotted for 30 min at room temperature (RT), and separated within 2 hours by sequential centrifugation (3,000 rpm, 10 min, RT; 16,000 rpm, 5 min, 4 °C). Hemolysis will be assessed visually and by 414 nm absorbance, with hemolyzed samples excluded. Serum will be stored at −80 °C and rechecked for hemolysis before testing. Total RNA will be extracted from serum using the miRNeasy Serum/Plasma Kit and quality-controlled by NanoDrop and Agilent Bioanalyzer Small RNA Kit. Small RNA libraries will be constructed using the TruSeq Small RNA Library Prep Kit (Illumina) and sequenced on the Illumina platform. Candidate miRNAs will be validated by RT-qPCR (miScript; QuantStudio 5).

High-throughput sequencing will be used to profile serum miRNAs in the experimental group (responders) and the control group (non-responders). Differentially expressed miRNAs between the two groups will be compared at baseline. MiRNAs with a fold change (FC) ≥2 or ≤0.5 and P < 0.05 will be defined as differentially expressed, where FC ≥2 indicates upregulation and FC ≤0.5 indicates downregulation. Among the miRNAs differentially expressed at baseline, we will further identify those that are differentially expressed between pre- and post-treatment in the experimental group but not in the control group. From these selected miRNAs, we will further screen for miRNAs that are differentially expressed between the two groups after treatment and define them as candidate miRNAs.

Subsequently, an additional 10 responders and 10 matched non-responders will be selected for RT-qPCR validation of the candidate miRNAs to further identify characteristic miRNAs associated with optimal response to acupuncture. Those with expression trends consistent with the high-throughput sequencing results, statistically significant between-group differences in expression, and fold changes reaching the preset threshold (FC ≥ 2 or ≤ 0.5) will be defined as target miRNAs.

### Value of serum miRNAs in predicting the effectiveness of acupuncture for DOR

The predictive value of target miRNAs for acupuncture effectiveness in patients with DOR will be analyzed using receiver operating characteristic (ROC) curves. With pre-treatment expression levels of target miRNAs as predictor variables and acupuncture effectiveness (responder vs. non-responder) as a binary outcome, the area under the ROC curve (AUC) and its 95% confidence interval, sensitivity, and specificity will be calculated. The optimal cutoff value for predicting acupuncture effectiveness will be determined by maximizing the Youden index, and the corresponding sensitivity and specificity will be reported.

### Sample size calculation

This study comprises two integrated components. The first is a prospective cohort study designed to evaluate the overall effectiveness of acupuncture. Patients receiving acupuncture treatment will be enrolled, with AFC as the primary outcome measure. Based on preliminary results, it was estimated that after 12 weeks of acupuncture, AFC would increase by 2.25 from baseline, with a standard deviation of 2.61 (22). Using the formula for a one-sample mean comparison (difference test) with a two-sided significance level of 5% and 80% power, the calculated sample size is 72 participants. Accounting for a 20% dropout rate, the required enrollment is set at 90 participants.

The second component is a nested case-control study embedded within this cohort to explore serum miRNA biomarkers. Based on previous findings, the response rate to acupuncture (defined as an increase in AFC of ≥2) is over 50%. To enable robust screening of differentially expressed miRNAs, we predefined a minimum requirement of 20 matched pairs (responders vs. non-responders) for sequencing. With a total cohort of 90 participants, and considering both the expected response rate and a 20% dropout rate, the cohort is projected to yield approximately 36 responders and 36 non-responders. This number far exceeds the pre-specified minimum requirement of 20 matched pairs, thereby providing an buffer against potential loss during the matching process. Consequently, the final sample size for the overall study is determined as 90 participants.

### Statistical analysis

Statistical analysis will be performed using IBM SPSS statistics 27. Missing data will be handled using multiple imputation. Continuous data will be presented as mean ± standard deviation or median with interquartile range. Categorical data will be presented as frequencies and percentages. For comparisons with baseline values, paired t-tests or Wilconxon signed-rank test will be used for continuous data, and rank sum tests for categorical data. For between-group comparisons, independent t-tests or Mann-Whitney U test will be used for continuous data as appropriate, and chi-square tests or Fisher’s exact test for categorical data. ROC curves will be employed to evaluate the predictive value of the screened miRNAs for acupuncture effectiveness in DOR, and the sensitivity, specificity, and AUC will be calculated. All statistical tests will be two-sided, and a P value < 0.05 will be considered statistically significant.

## Discussion

This study protocol describes a prospective nested case-control study designed to identify serum miRNA expression profiles capable of distinguishing DOR patients who respond favorably to acupuncture from those who do not. To our knowledge, this is the first clinical study to explore the utility of circulating miRNAs as predictive biomarkers for acupuncture response in this population. The findings are expected to provide a molecular basis for the precise selection of acupuncture-responsive individuals, thereby addressing a critical unmet need in personalized fertility care.

Accumulating clinical evidence supports the potential of acupuncture to improve ovarian function, as reflected by significant increases in AFC and reductions in basal FSH levels (11). A systematic review and meta-analysis including 11 RCTs with a total of 885 DOR patients showed that acupuncture combined with IVF significantly improved clinical pregnancy and live birth rates (12). However, marked heterogeneity in treatment response poses a substantial challenge to clinical decision-making. While some patients achieve meaningful reproductive improvements, others derive little or no benefit despite undergoing identical, prolonged, and costly treatment protocols. This variability underscores the urgent need for reliable predictive tools that can identify likely responders prior to or early during the treatment course.

Currently, predictive models for acupuncture effectiveness in DOR remain scarce, and most existing efforts have relied on conventional clinical indicators such as age, AMH, FSH, AFC, and FSH/LH ratio. Although some of these models have demonstrated modest predictive value (23), they essentially reflect the current functional reserve of the ovaries rather than the individual’s molecular responsiveness to acupuncture. This intrinsic limitation may explain why patients with comparable baseline profiles exhibit divergent treatment outcomes. Thus, there is an urgent need to identify novel molecular biomarkers that can complement and extend the predictive capacity of traditional clinical parameters.

MiRNAs have emerged as promising predictive biomarkers due to their stability in peripheral blood and their potential regulatory roles in ovarian physiology, including folliculogenesis, granulosa cell apoptosis, and hormone metabolism. Studies have shown that miRNAs are widely involved in follicular development, oocyte maturation, and sex hormone synthesis (24), and that aberrant miRNA expression is associated with ovarian aging and premature ovarian failure (25, 26). Notably, differential miRNA expression profiles have been observed among women with low, normal, and high ovarian reserve, with certain miRNAs correlating with AMH levels, suggesting that circulating miRNAs may serve as auxiliary markers of ovarian reserve status (27). At the mechanistic level, miR-484 is highly expressed in granulosa cells of DOR patients and is associated with decreased AMH and AFC as well as elevated FSH, potentially through targeting YAP1, inducing mitochondrial dysfunction, and promoting granulosa cell apoptosis (28). Similarly, miR-23a and miR-27a promote human granulosa cell apoptosis via SMAD5 targeting and activation of the FasL-Fas pathway (29). These observations suggest that miRNAs, as upstream regulators of gene expression, may reflect individual molecular responsiveness to acupuncture, and their differential expression may be directly linked to the signaling pathways through which acupuncture exerts its ovarian protective effects. Thus, baseline serum miRNA profiles hold promise for “pre-treatment prediction” of acupuncture efficacy, potentially distinguishing patients who are inherently suitable for acupuncture from those who are not. Beyond biomarkers discovery, this approach may also offer mechanistic insights into the molecular basis of acupuncture’s beneficial effects on ovarian function.

The adoption of a nested case-control design represents a major methodological strength of this protocol. By first establishing a prospective cohort of DOR patients receiving standardized acupuncture treatment, we are able to objectively assess treatment response using both single-case effectiveness criteria and overall outcome measures. The 1:1 matching of non-responders to responders based on key baseline prognostic factors, including age, AMH, AFC, and FSH levels, helps minimize confounding, thereby enhancing the internal validity of the comparison. Furthermore, the two-stage approach incorporating high-throughput sequencing for discovery and RT-qPCR for validation ensures that the identified miRNA candidates are robust and reproducible, a prerequisite for any potential clinical translation.

Nevertheless, as a discovery-phase investigation rather than a validation study, this work has several limitations that warrant careful consideration. First, this is a single-center study with a relatively limited sample size for the nested case-control comparison, which may limit the generalizability and fail to capture the full spectrum of miRNA diversity among DOR patients. Second, the absence of a sham acupuncture or no-treatment control group precludes the unequivocal attribution of observed miRNA changes to acupuncture per se, as they may partly reflect natural fluctuations or placebo-related effects. Third, although the 12-week treatment period is adequate for detecting changes in AFC and hormone levels, it may be insufficient to evaluate long-term reproductive outcomes such as clinical pregnancy and live birth. Fourth, although clinical predictors will be systematically collected, we have not yet constructed a combined model integrating miRNA markers with clinical indicators; the incremental predictive value of miRNAs over traditional clinical measures therefore remains to be established. Finally, the findings of this study should be interpreted as exploratory, and external validation in an independent, large-scale prospective cohort is necessary to evaluate the generalizability of its findings across diverse responder types in real-world clinical settings.

In conclusion, this study is expected to provide preliminary but valuable evidence regarding the utility of serum miRNAs as predictive biomarkers for acupuncture effectiveness in DOR. Future large-scale, prospective cohort are warranted to validate and extend our findings. Integrating miRNA biomarker profiling into clinical practice may advance personalized reproductive medicine by enabling more targeted and effective management strategies for DOR patients.
